# Auction market placement and a rest stop during transportation affect the respiratory bacterial microbiota of beef cattle

**DOI:** 10.3389/fmicb.2023.1192763

**Published:** 2023-09-22

**Authors:** Muhammed Salah Uddin, Karen S. Schwartzkopf-Genswein, Matthew Waldner, Daniela M. Meléndez, Yan D. Niu, Trevor W. Alexander

**Affiliations:** ^1^Lethbridge Research and Development Centre, Agriculture and Agri-Food Canada, Lethbridge, AB, Canada; ^2^Department of Agricultural, Food and Nutritional Science, University of Alberta, Edmonton, AB, Canada; ^3^Faculty of Veterinary Medicine, University of Calgary, Calgary, AB, Canada

**Keywords:** auction market, bovine respiratory disease, rest stops, transportation, respiratory microbiota

## Abstract

**Background:**

Bovine respiratory disease (BRD) is a significant health problem in beef cattle production, resulting in considerable economic losses due to mortalities, cost of treatment, and reduced feed efficiency. The onset of BRD is multifactorial, with numerous stressors being implicated, including transportation from farms to feedlots. In relation to animal welfare, regulations or practices may require mandatory rest times during transportation. Despite this, there is limited information on how transportation and rest stops affect the respiratory microbiota.

**Results:**

This study evaluated the effect of cattle source (ranch-direct or auction market-derived) and rest stop duration (0 or 8 h of rest) on the upper respiratory tract microbiota and its relationship to stress response indicators (blood cortisol and haptoglobin) of recently weaned cattle transported for 36 h. The community structure of bacteria was altered by feedlot placement. When cattle were off-loaded for a rest, several key bacterial genera associated with BRD (*Mannheimia*, *Histophilus*, *Pasteurella*) were increased for most sampling times after feedlot placement for the ranch-direct cattle group, compared to animals given no rest stop. Similarly, more sampling time points had elevated levels of BRD-associated genera when auction market cattle were compared to ranch-direct. When evaluated across time and treatments several genera including *Mannheimia, Moraxella, Streptococcus and Corynebacterium* were positively correlated with blood cortisol concentrations.

**Conclusion:**

This is the first study to assess the effect of rest during transportation and cattle source on the respiratory microbiota in weaned beef calves. The results suggest that rest stops and auction market placement may be risk factors for BRD, based solely on increased abundance of BRD-associated genera in the upper respiratory tract. However, it was not possible to link these microbiota to disease outcome, due to low incidence of BRD in the study populations. Larger scale studies are needed to further define how transportation variables impact cattle health.

## Introduction

Bovine respiratory disease (BRD), also called shipping fever, remains the most common cause of morbidity and mortality after feedlot placement (Booker et al., 2008), resulting in significant economic losses (Timsit et al., 2016a). It is a multifactorial disease but bacterial species, including *Histophilus somni*, *Mannheimia haemolytica*, *Mycoplasma bovis*, and *Pasteurella multocida* are frequently implicated (Confer, 2009). The upper respiratory tract is a reservoir of these opportunistic pathogens, which can proliferate and infect the lungs when cattle immunity is compromised due to stress and/or primary viral infections (Hodgson et al., 2005). High risk cattle (recently weaned, commingled at auction market, transported for a longer period of time, unknown immunization history) populations entering feedlots are most susceptible to BRD. As a result, these cattle are often administered metaphylactic antibiotics to reduce respiratory infections after feedlot placement. However, an increase in the prevalence of antimicrobial-resistant BRD pathogens from cattle emphasizes a need for alternatives to disease management, including potentially altering management practices related to beef production.

Several stressors have been implicated in the pathogenesis of BRD including abrupt weaning, transportation, sale through an auction barn, commingling, surgical procedures at entrance to the feedlot, feed adaptation, and adverse environmental conditions (Taylor et al., 2010). All these stressors may increase blood cortisol concentrations, which in turn, can impair host defenses and reduce containment of pathogens in the upper respiratory tract (Rice et al., 2007). For example, transportation and cold temperatures transiently elevated plasma cortisol concentrations (Filion et al., 1984; Slocombe et al., 1984). Interestingly, neurochemicals secreted during stressful events can potentially influence the composition of the respiratory tract microbiota, as these neurochemicals can interact with bacterial pathogens, modifying their growth and expression of virulence-related factors (Lyte, 2014). Corticotropin-releasing hormone, a peptide hormone involved in the stress response, enhanced growth and virulence of the human respiratory pathogen *Streptococcus pneumoniae*, and is thought to be involved in its transition from a non-pathogenic to a pathogenic state (Ndjom and Jones, 2015). In cattle, the abundance of *M. haemolytica* was markedly increased in recently weaned calves that were transported > 1,600 km (Frank and Smith, 1983), perhaps also suggesting that the microbiota of the bovine respiratory tract respond to stress factors. However, the extent to which transportation stress may affect respiratory pathogenic and commensal bacteria is not fully known, as there are no controlled studies that have evaluated the relationship.

Safe and humane transportation of livestock carries important public and trade concerns worldwide due to their impacts on animal health and welfare, food quality and safety, and economics (Harris, 2005). The recently revised transport regulations for Canada indicate weaned and unweaned calves cannot be confined on a truck for more than 36 and 12 h (H), respectively, without being provided food, water and rest for a minimum of 8H. The association between rest stop duration and cattle health and welfare outcomes is, however, not well studied scientifically. Transportation has been known to result in stress and injury to livestock [Food and Agriculture Organization (FAO), 2002]. Factors contributing to transport stress include the duration of the journey, feed and water withdrawal, fatigue associated with lying deprivation, increased energy expenditure to maintain balance, highly variable environmental conditions (Fisher et al., 2005), and in some cases the stress of weaning (Schwartzkopf-Genswein and Grandin, 2019). Recent studies have indicated that the stress responses associated with long distance transport could be reduced by the provision of a resting period where animals can lie down and are given access to feed and water (Cooke et al., 2013).

Whether host stress impacts the respiratory microbiota is highly relevant to animal health and predisposition to BRD, especially if stress factors promote pathogen dominance over commensal bacteria. Establishment and stability of the resident microbiota of the respiratory tract are critical in colonization and resistance against pathogens (Bogaert et al., 2004; Cho and Blaser, 2012), with microbial disruption potentially predisposing to pathogenesis (Krismer et al., 2017). We have previously shown that there is an abrupt shift in the nasopharyngeal (NP) microbiota of cattle upon entry to a feedlot (Holman et al., 2017). However, in that study (Holman et al., 2017), whether this shift of microbiota was attributed to transportation remains elusive. In addition, associations between host stress factors and the respiratory microbiota under natural conditions have not been studied. Novel information relating to the impact of transportation and rest stop duration could lead to microbiota-based interventions, including management practices related to transportation, that provide new opportunities for managing cattle in place of antibiotics. The objective of this study was to determine the relationship between indicators of stress (cortisol and haptoglobin) and the respiratory microbiota in beef calves sourced from a ranch or auction market, and transported with and without a rest stop.

## Methods

This protocol was approved by the Animal Care Committee of Lethbridge Research and Development Centre (Animal Use Protocol number 1918). Animals were cared for in according with the Canadian Council of Animal Care (The CCAC, 2009).

### Study design and sampling

This study was part of a larger study assessing the effect of transport and rest stops on the health and welfare of beef calves (Meléndez et al., 2021). One hundred sixty crossbred steer calves (Black or Red Angus × Hereford/Simmental and Black or Red Angus × Charolais) were sourced from a single farm in southern Alberta and transported 1H to the Lethbridge Research and Development Centre (LeRDC), as described previously (Meléndez et al., 2021). Calves were randomly divided into two groups: those shipped to an auction market prior to transportation (Auction Market, AM), or those directly transported without auction market placement (Ranch). Within each group, calves were randomly assigned to two rest stop times, which included 0H or 8H of rest, following 36H of transportation. These four treatment groups are thus labeled by source and rest time: AM-0H, AM-8H, Ranch-0H, and Ranch-8H. There were 40 calves in each treatment group, housed in 4 pens (21 × 27 m); 10 animals per pen. Three calves per pen were randomly selected for physiological and microbial parameters to be repeatedly measured, for a total of 12 calves/treatment.

The Auction Market calves were transported for approximately 20 min to a local auction market, 7.9 km from the Lethbridge Research and Development Centre’s feedlot. Calves were off-loaded and sorted into pens with access to hay and water. Calves spent 24H at the auction market and within that time, they were moved through the sale ring to mimic auction market conditions. The calves were not comingled with calves from other farms, and were transported for 36H after the 24H period in the auction market. Although commingling is considered one of the important risk factors for BRD (Smith, 2020), this current study did not follow this practice to assess the stress caused by handling and transport alone. Calves were loaded onto trailers at the auction market and LeRDC feedlot on the same day, at the same time. Calves were transported for 36H, followed by the assigned rest stop time (0 or 8 H), and transported another 4H for delivery to the LeRDC feedlot (Supplementary Figure 1).

After the additional 4H transport, calves were unloaded and processed, which included: a 7-way bovine clostridial vaccine (Ultrabac/Somubac, Zoetis Canada Inc., Kirkland, Quebec, Canada); a 5-way bovine viral diarrhea, rhinotracheitis, parainfluenza and bovine respiratory syncytial virus vaccine (Pyramid FP 5 + Presponse SQ, Boehringer Ingelheim, Burlington, Ontario, Canada); an antibiotic (Draxxin, Zoetis Canada Inc., Kirkland, Canada); and an anti-parasitic agent (Ivomec Pour-on for Cattle, Boehringer Ingelheim, Burlington, Ontario, Canada). Calves were sampled (N = 12 per treatment) before loading for transportation (BL), after transportation and unloading at the feedlot (AU), 1 day, 3 days, 6 days, and 28 days AU. Blood and NP samples were collected at each sampling time. Blood samples were collected and processed for cortisol and haptoglobin, as described by Meléndez et al. (2021). NP swabs were collected from the right nasal cavity immediately after blood collection while calves were restrained in a chute. Prior to sampling, the nostril was wiped clean with 70% ethanol. Extended guarded swabs (27 cm) with a rayon bud (MW 124, Medical Wire & Equipment, Corsham, England) were used for sampling (Holman et al., 2017). Swab tips were then cut and placed into sterile 1.5 mL tubes kept on ice. Samples were transported to the lab and stored at −80°C within 1 h of collection. Throughout the study, two calves became sick during feedlot placement. Both were cases of fever; one within the AM-8H treatment group, and one within the Ranch-8H treatment group.

### Physiological and microbial analyses

Blood cortisol and haptoglobin were quantified and reported by Meléndez et al. (2021). Briefly, cortisol levels were quantified from serum using an immunoassay kit (DetectX Kit, Arbor Assays, Ann Arbor, MI, United States) while a colorimetric assay (Tridelta Development Ltd., Maynooth, County Kildare, Ireland) was conducted to quantify the serum haptoglobin concentrations. Total nucleic acids were extracted from NP swabs as described previously (Holman et al., 2017). In DNA from all swabs, the V4 region of the 16S rRNA gene was amplified using primers 515-F (5′-GTGYCAGCMGCCGCGGTAA-′3) and 806-R (5′-GGACTACNVGGGTWTCTAAT-′3; Holman et al., 2019). The amplicon was sequenced on a MiSeq instrument (PE250, with 25,000 read coverage, Genome Quebec, Montreal, Quebec, Canada) with the MiSeq Reagent Kit v2.

For the V4 amplicons, quality verification and summarization of raw reads were performed with FastQC 0.11.9 and MultiQC 1.12 (Ewels et al., 2016). Sequence trimming was performed with Trimmomatic 0.39 (Bolger et al., 2014) to remove primers and low-quality read sections with settings HEADCROP:18, SLIDINGWINDOW:5:18, LEADING:3, and TRAILING:3. Further statistical analysis was performed in R 4.1.0 (R Core Team, 2022). The trimmed paired reads were additionally filtered with DADA2 1.22.0 (Callahan et al., 2016) filterAndTrim with default settings and merged. Bimeric sequences were removed from the merged sequence set with DADA2 removeBimeraDenovo. Taxonomic classification of the 16S sequences was performed with DADA2 IdTaxa using the SILVA 138 database (Quast et al., 2013) to construct an amplicon sequence variants (ASVs) table. As SILVA 138 is the most recent and readily used 16S classification database but lacks recent updates to the taxonomic database, ASVs classified with the genus “*Mycoplasma*” have had their taxonomy manually updated to current standards. Phyloseq 1.38.0 (McMurdie and Holmes, 2013), vegan 2.5–7 (Oksanen et al., 2019), and DESeq2 1.34.0 (Love et al., 2014) were used for statistical analysis of the data and plots were visualized with ggplot2 3.3.5 (Wickham, 2011). Alpha diversity of the dataset was assessed with the Shannon diversity index and richness (observed ASVs) through vegan, and plotted. The ASV table was filtered for ASVs present (count ≥ 2) in at least 1% of the samples to reduce noise in downstream analysis. A 1-way ANOVA was used to evaluate alpha diversity with respect to treatment and time. Microbial community structure was analyzed with vegan using permutational multivariate analysis of variance (PERMANOVA; Adonis function) with 9,999 permutations to determine the effect of treatment at each sampling time. To calculate beta diversity, the filtered ASV counts were normalized with size factors calculated with GMPR in DESeq2. Sample-sample distances were determined with the Bray-Curtis metric using phyloseq ordinate and visualized with detrended correspondence analysis (DCA). The phyla and genus with the highest abundance in each treatment and sample time were identified with phyloseq and visualized. Significant [*p* < 0.05, log2(FC) > 2 or log2(FC) < −2] differentially abundant genera were identified with DESeq2 by fitting a negative binomial model to two separate equations, sampling time X vs. sampling time BL (~Time), and sampling time X vs. sampling time BL with respect to treatment time points vs. the first sampling time point BL (“~Treatment + Time + Time:Treatment”). Significant ASVs (*p* < 0.05) within the significant genera were visualized with heatmaps, both within the genera and with respect to individually significant ASVs. Correlation analysis of cortisol and haptoglobin levels sampled across all treatments and time points were performed against the top 9 most abundant genera at the genera and ASV taxonomic levels using phylosmith 1.0.6 (Smith, 2019). Correlation analysis utilized the Spearman’s rank correlation coefficient (r_s_) to assess the relationship between the variables.

## Results

### 16S rRNA gene sequencing overview

The raw ASV table contained 10,756 ASVs with a total of 9,668,873 merged paired reads assigned to 276 samples (data not shown). The median number of sequences per sample was 35,172.5 ± 14,376.4 with a minimum of 45 and maximum of 70,203. After filtering, the ASV table contained 1,097 SVs with a total of 9,022,362 merged paired reads. The median number of sequences per sample was 33,701 ± 14,982.9 with a minimum of 2 and maximum of 70,065.

### The community structure of the NP microbiota

**Figure 1:**
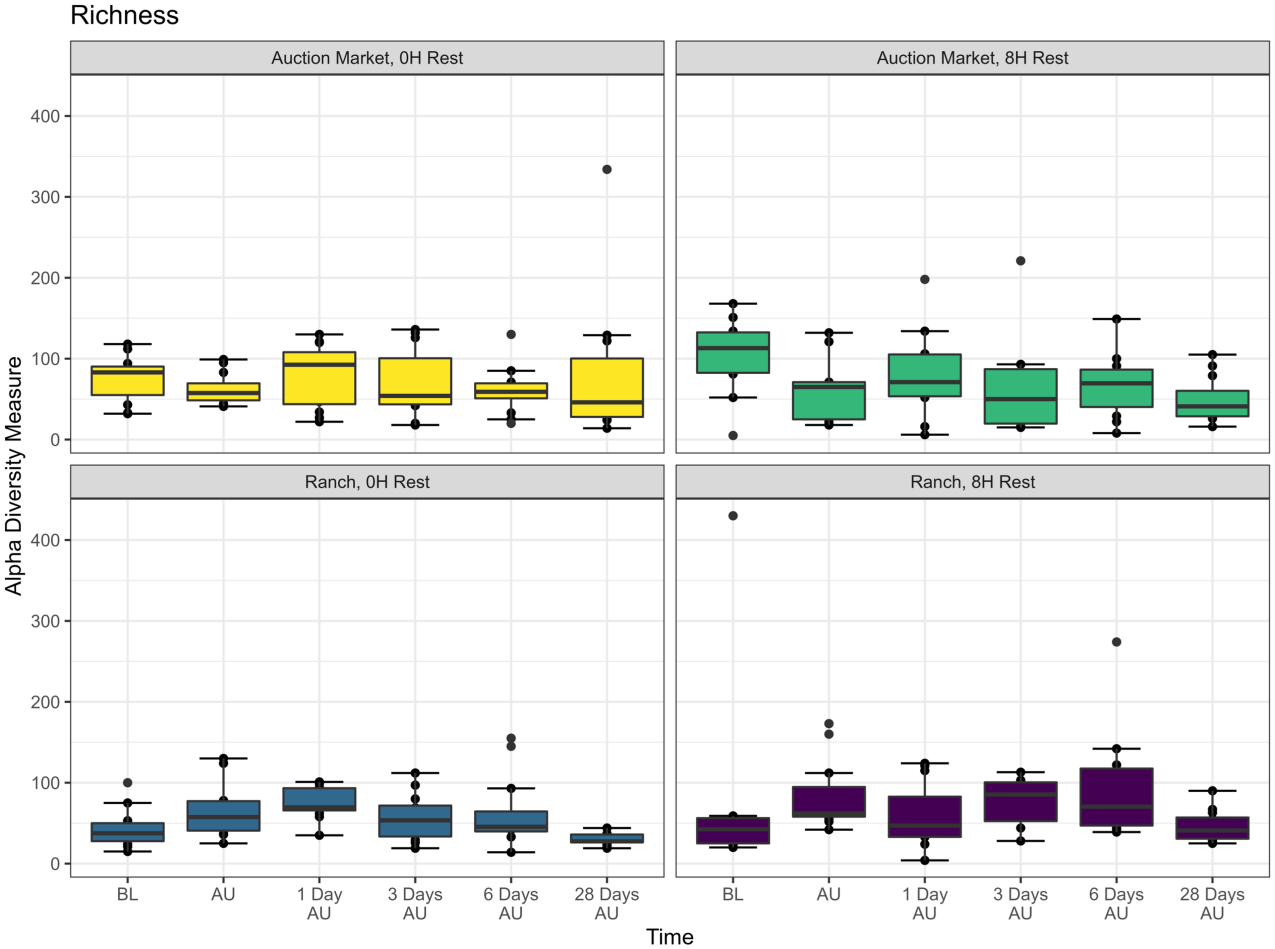
Alpha diversity showing bacterial Richness (counts of taxa observed) of nasopharyngeal samples. Calves were sourced from the same farm and either placed in an auction market (Auction Market) prior to transportation or were directly received from the farm (Ranch). Within each group, calves were assigned to rest (8H) and no rest (0H) treatments, following 36H of transportation. Subsequent to the rest interval, calves were transported for an additional 4H prior to unloading at a feedlot. Nasopharyngeal samples were collected before loading for transportation (BL), after unloading at the feedlot (AU), and 1 day AU, 3 days AU, 6 days AU, and 28 days AU. Error bars indicate ± standard error of the mean. The box in the plots indicates the interquartile range (IQR; middle 50% of the data), the middle line represents the median value, and the whiskers represents 1.5 times the IQR.

**Figure 2:**
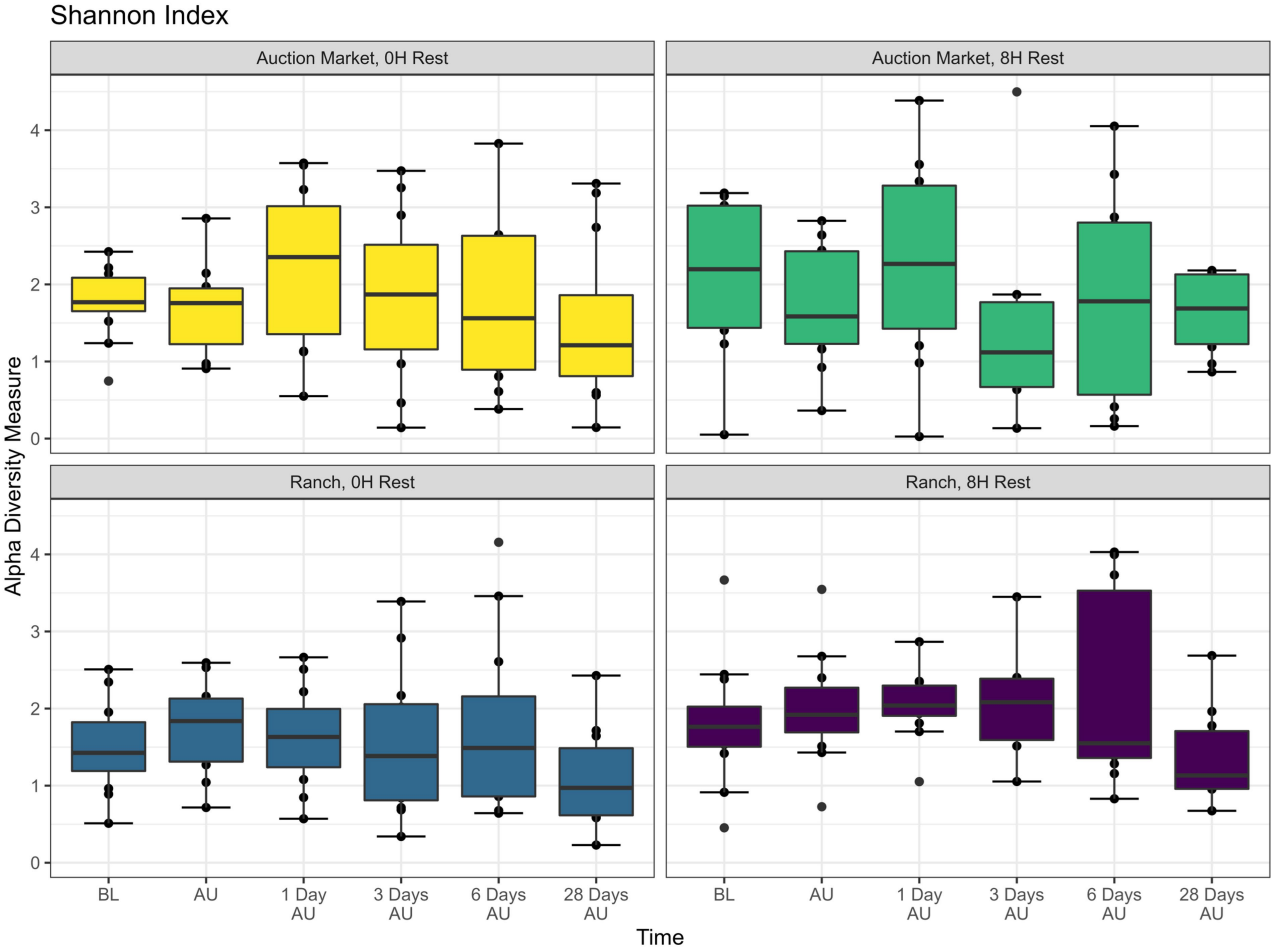
Alpha diversity showing Shannon Index of nasopharyngeal samples. Calves were sourced from the same farm and either placed in an auction market (Auction Market) prior to transportation or were directly received from the farm (Ranch). Within each group, calves were assigned to rest (8H) and no rest (0H) treatments, following 36H of transportation. Subsequent to the rest interval, calves were transported for an additional 4H prior to unloading at a feedlot. Nasopharyngeal samples were collected before loading for transportation (BL), after unloading at the feedlot (AU), and 1 day AU, 3 days AU, 6 days AU, and 28 days AU. Shannon diversity takes into consideration the number of genera and the proportion of each genus in a sample. Error bars indicate ± standard error of the mean. The box in the plots indicates the interquartile range (IQR; middle 50% of the data), the middle line represents the median value, and the whiskers represents 1.5 times the IQR.

**Figure 3:**
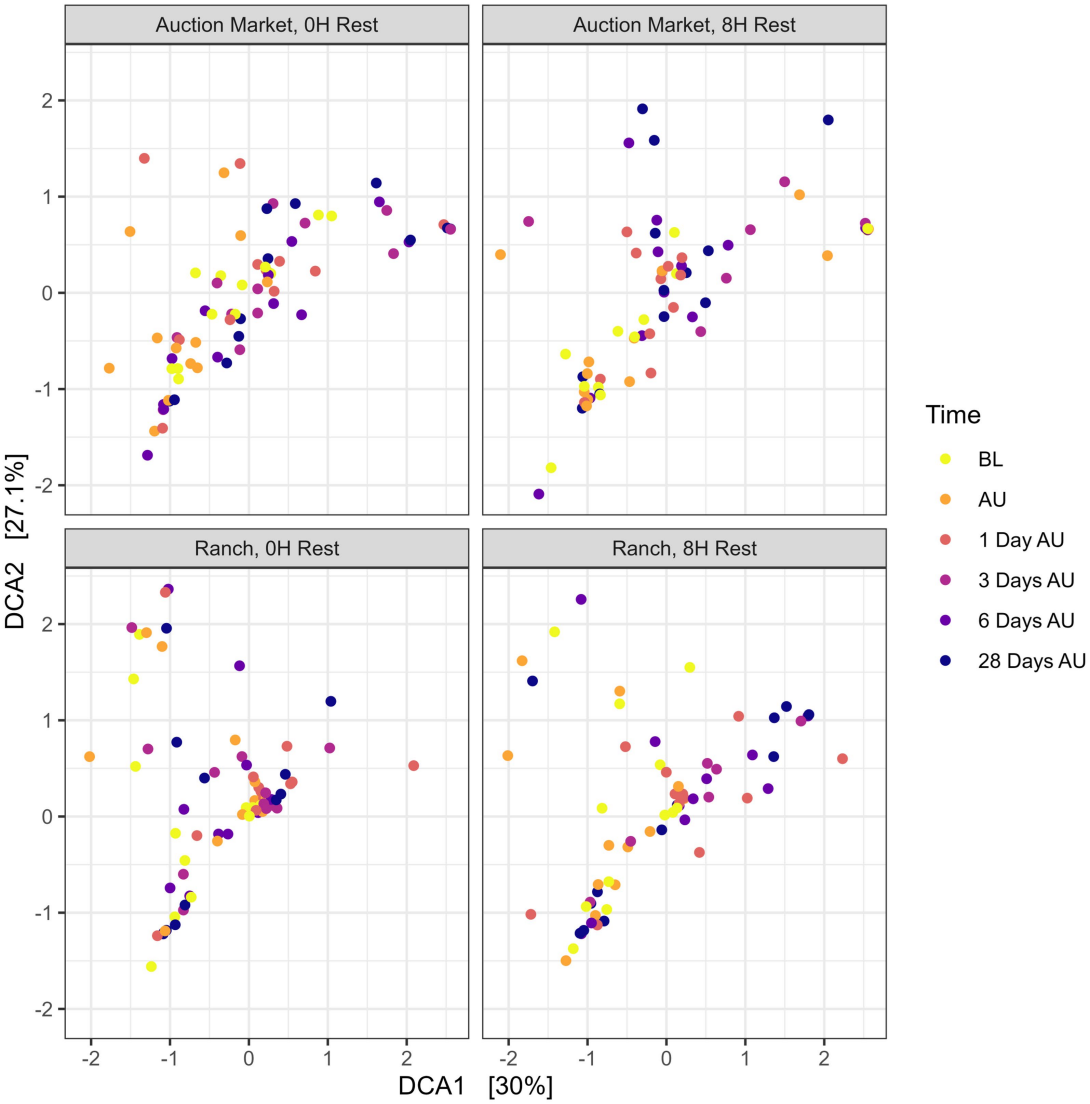
Detrended correspondence analysis (DCA) of the Bray-Curtis metric showing clustering of each treatment over time for the nasopharyngeal samples. Calves were sourced from the same farm and either handled through an auction market (Auction Market) prior to transportation or were obtained directly from the farm (Ranch). Within each group, calves were assigned to rest (8H) and no rest (0H) treatments, following 36H of transportation. Subsequent to the rest interval, calves were transported for an additional 4H prior to unloading at a feedlot. Nasopharyngeal samples were collected before loading for transportation (BL), after unloading at the feedlot (AU), and 1 day AU, 3 days AU, 6 days AU, and 28 days AU.

The source of the calves, the combination of source and rest time as treatments (AM-0H, AM-8H, Ranch-0H, and Ranch-8H), and the interaction between the treatments and the sampling time of the cattle did not affect (*p* > 0.05) either richness (Figure 1) or Shannon diversity (Figure 2) metrics. However, Shannon diversity was generally increased at individual time points for Ranch-sourced cattle provided 8 H of rest compared to those provided no rest (Figure 2; *p* < 0.05). PERMANOVA revealed that bacterial structure of the microbiota was significantly affected by the source of the calves (*p* < 0.001, R^2^ = 0.019), the treatment (source and rest time combined, *p* < 0.001, R^2^ = 0.030), and the sampling time (*p* < 0.001, R^2^ = 0.060). The bacterial structure of the microbiota tended to be affected by rest time alone (*p* < 0.1). DCA plots (Figure 3) displayed the variance in microbiota structure between both sampling time and treatments for the samples. In support of the PERMANOVA results, variation between treatments was observed via the differences in the spread in each subplot. The locations of samples with different sampling times also varied.

### Composition of the NP microbiota

**Figure 4:**
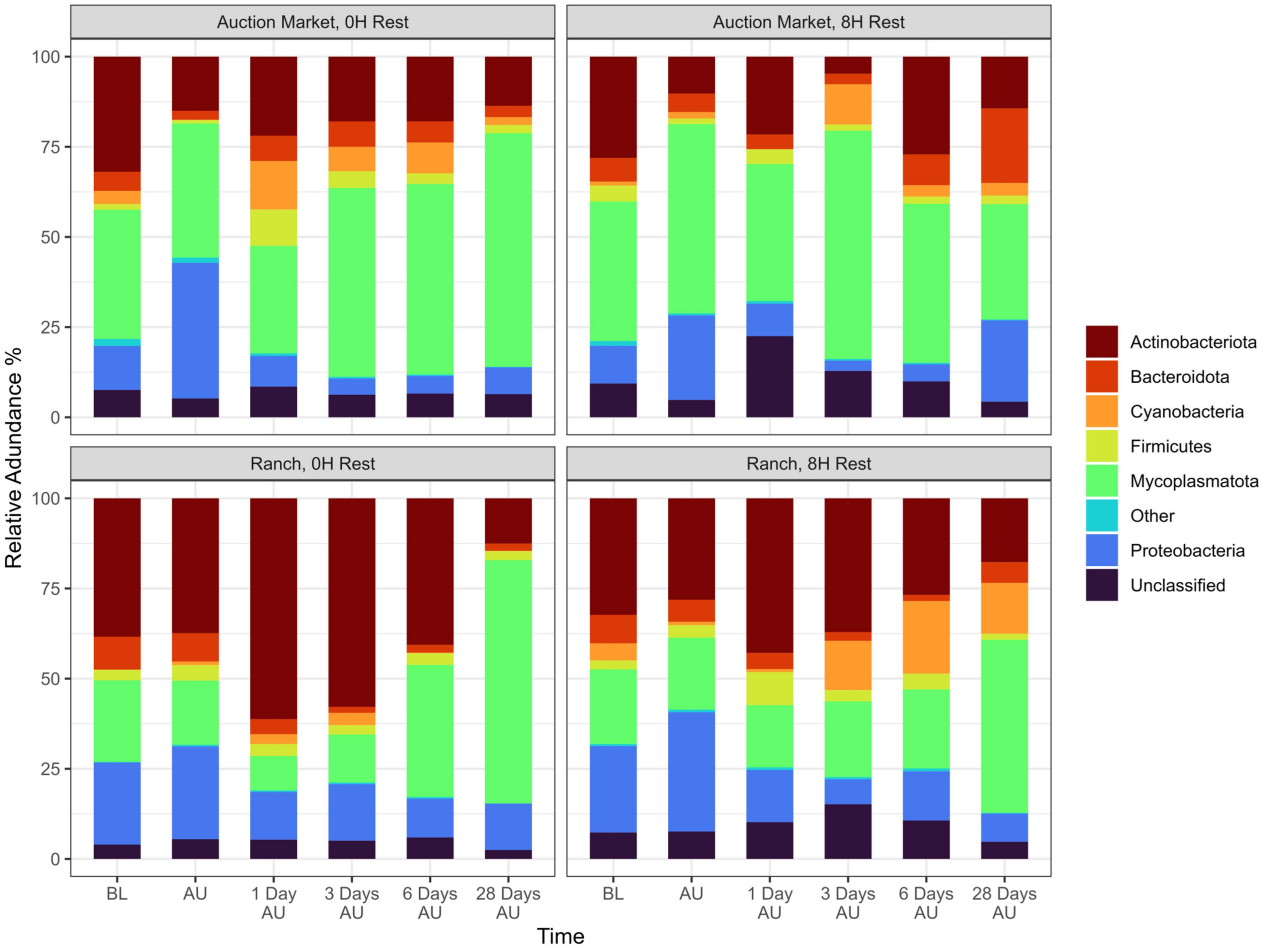
Relative abundance of 16S sequences for the six most abundant phyla from nasopharyngeal samples taken at each treatment and time. Calves were sourced from the same farm and either placed in an auction market (Auction Market) prior to transportation or were directly received from the farm (Ranch). Within each group, calves were assigned to rest (8H) and no rest (0H) treatments, following 36H of transportation. Subsequent to the rest interval, calves were transported for an additional 4H prior to unloading at a feedlot. Nasopharyngeal samples were collected before loading for transportation (BL), after unloading at the feedlot (AU), and 1 day AU, 3 days AU, 6 days AU, and 28 days AU. 16S sequences that were assigned to a phylum lacking enough abundance to be visualized were assigned within the “Other” bar. The “Unclassified” bar represents the relative abundance of 16S sequences that were unable to be assigned a phylum level.

**Figure 5:**
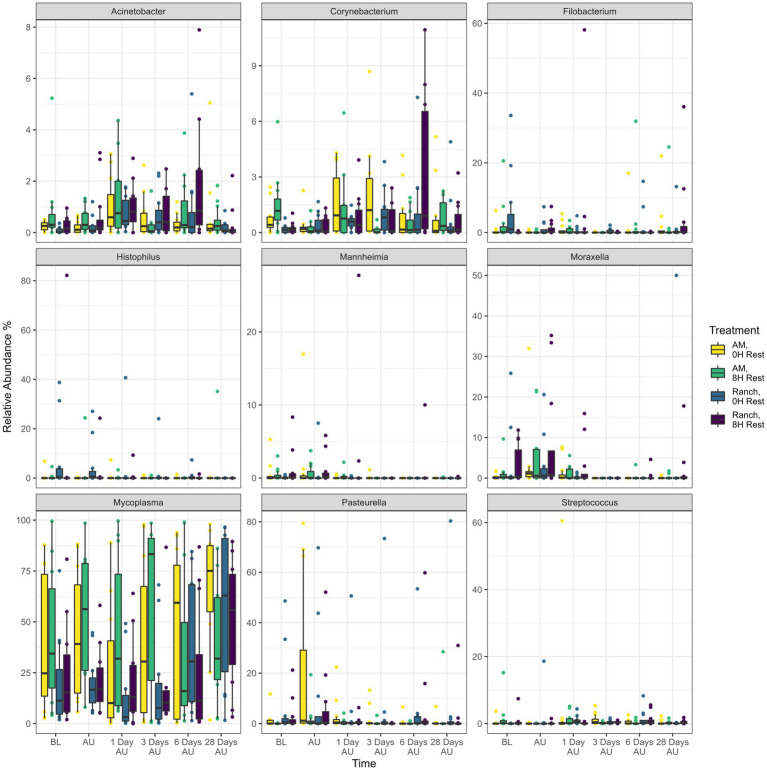
Relative abundance of the nine most abundant genera identified in nasopharyngeal swabs for each treatment and time. Calves were sourced from the same farm and either placed in an auction market (Auction Market) prior to transportation or were directly received from the farm (Ranch). Within each group, calves were assigned to rest (8H) and no rest (0H) treatments, following 36H of transportation. Subsequent to the rest interval, calves were transported for an additional 4H prior to unloading at a feedlot. Nasopharyngeal samples were collected before loading for transportation (BL), after unloading at the feedlot (AU), and 1 day AU, 3 days AU, 6 days AU, and 28 days AU. Error bars indicate ± standard error of the mean. The box in the plots indicates the interquartile range (IQR; middle 50% of the data), the middle line represents the median value, and the whiskers represents 1.5 times the IQR.

Across sampling time and treatment groups, a total of 18 different bacterial phyla were identified, among which Mycoplasmatota (38.5%), Actinobacteriota (29.2%), Proteobacteria (16.5%), Bacteroidota (6.1%), Cyanobacteria (4.7%), and Firmicutes (3.6%) were the most relatively abundant, and together constituted 98.6% of the sequences (Figure 4). The diversity of genera within each phylum varied with the relative abundance of a single genus ranging from <1% to 100% of a phylum. Overall, the 9 most relatively abundant genera across treatments and time included *Mycoplasma* (38.5%), *Pasteurella* (5.0%), *Histophilus* (1.7%), *Moraxella* (1.6%), *Filobacterium* (1.3%), *Streptococcus* (0.7%), *Corynebacterium* (0.6%), *Acinetobacter* (0.5%), and *Mannheimia* (0.4%; Figure 5).

### Changes in the microbiota across sampling time and between treatments

**Figure 6:**
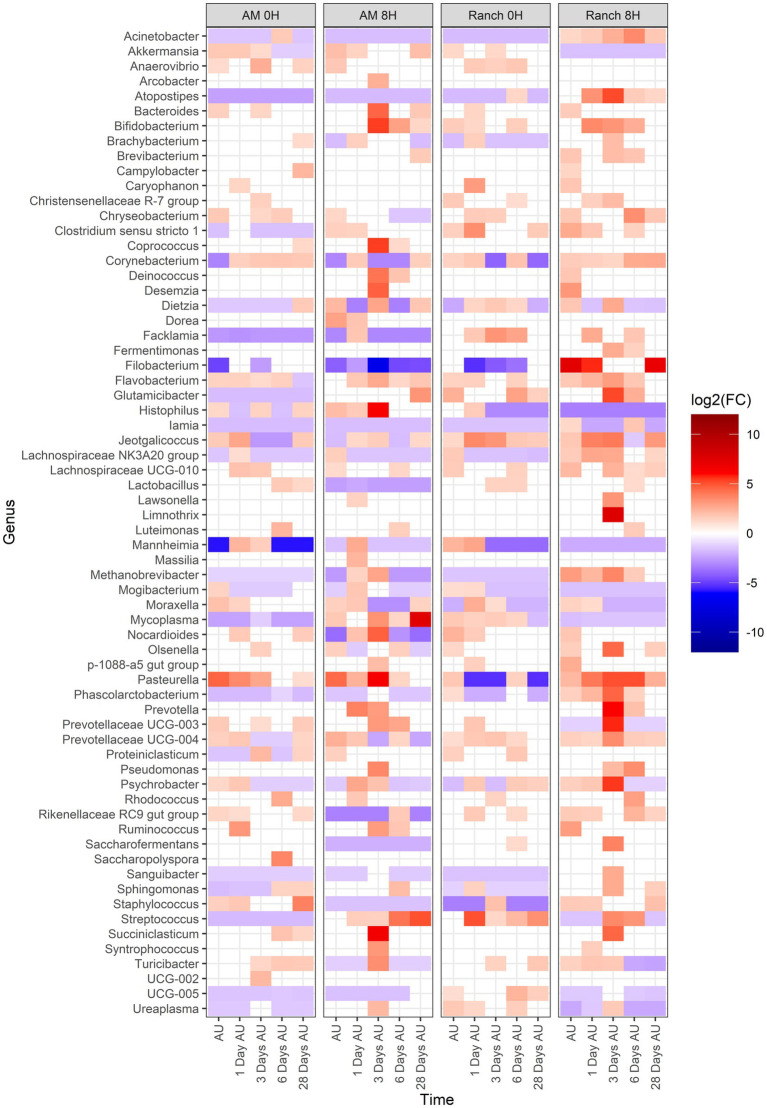
Genera that showed a significant change [*p* < 0.05, log2(FC) > 2 or log2(FC) < −2] of each sampling time against a baseline time (Before Loading) within the treatments. The colors displayed represent the average log2(FC) of amplicon sequence variants (ASVs) with a significant change (*p* < 0.05) within the respective genus at the indicated time. Calves were sourced from the same farm and either placed in an auction market (AM) prior to transportation or were directly received from the farm (Ranch). Within each group, calves were assigned to rest (8H) and no rest (0H) treatments, following 36H of transportation. Subsequent to the rest interval, calves were transported for an additional 4H prior to unloading at a feedlot. Nasopharyngeal samples were collected before loading for transportation, after unloading at the feedlot (AU), and 1 day AU, 3 days AU, 6 days AU, and 28 days AU.

Across all treatments, 66 genera changed [*p* < 0.05, log2(FC) > 2 or log2(FC) < −2] in relative abundance from the BL sampling time (Figure 6). Of these, 51, 58, 47, and 58 genera showed changes in relative abundance for the AM-0H, AM-8H, Ranch-0H, and Ranch-8H treatments, respectively. All of the 9 most relatively abundant genera were included in these taxa that changed across time. Interestingly, *Acinetobacter*, *Atopostipes*, and *Filobacterium* were consistently less abundant in AM-0H, AM-8H, and Ranch-0H treatments, but were elevated at all time points for Ranch-8H calves. *Histophilus* was strongly elevated at 3 days AU in the AM-8H calves but was reduced at most time points for Ranch-0H and Ranch-8H calves. In contrast, *Pasteurella* increased at most time points compared to the base line for AM-0H, AM-8H, and Ranch-8H calves, while it was lower at 3 out of 5 time points for the Ranch-0H calves.

**Figure 7:**
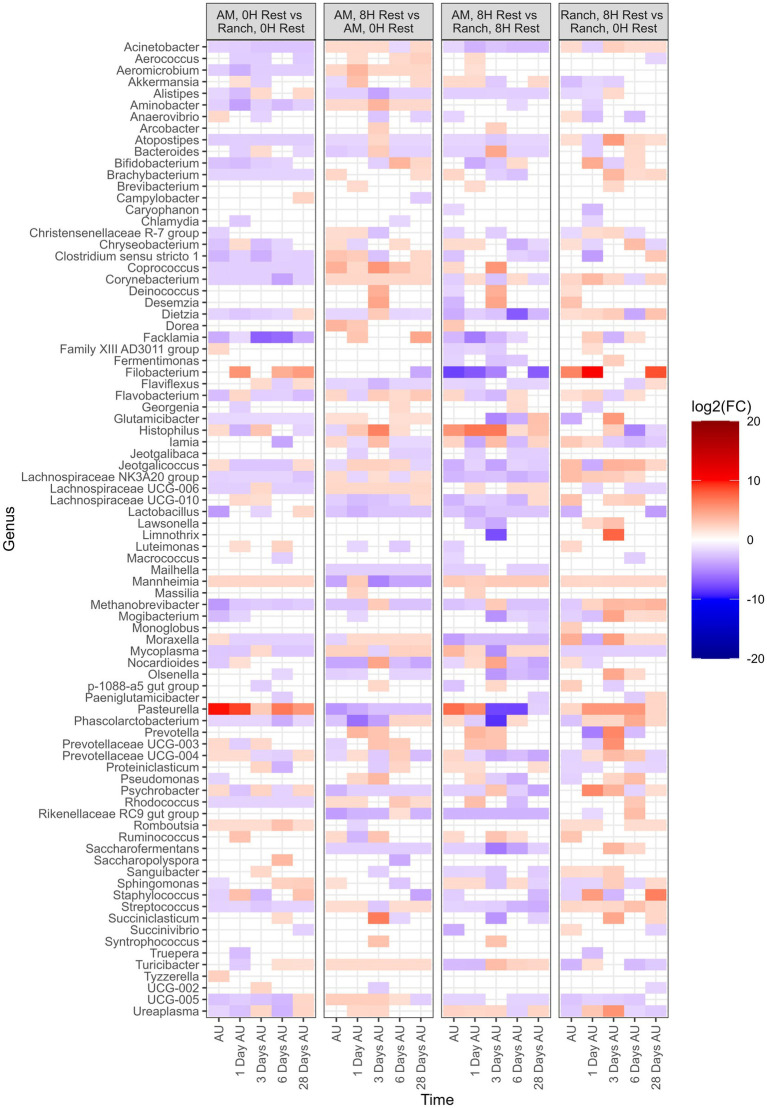
Genera of nasopharyngeal swabs that showed a significant change [*p* < 0.05, log2(FC) > 2 or log2(FC) < −2] between noted treatments, with respect to each sampling time against the baseline time (Before Loading). The colors displayed represent the average log2(FC) of amplicon sequence variants (ASVs) with a significant change (*p*  < 0.05) within the respective genus at the indicated time. Calves were sourced from the same farm and either placed in an auction market (AM) prior to transportation or were directly received from the farm (Ranch). Within each group, calves were assigned to rest (8H) and no rest (0H) treatments, following 36H of transportation. Subsequent to the rest interval, calves were transported for an additional 4H prior to unloading at a feedlot. Nasopharyngeal samples were collected before loading for transportation, after unloading at the feedlot (AU), and 1 day AU, 3 days AU, 6 days AU, and 28 days AU. The treatment vs. treatment comparisons are as follows: “Auction Market, 0H Rest” vs. “Ranch, 0H Rest”, “Auction Market, 8H Rest” vs. “Auction Market, 0H Rest”, “Auction Market, 8H Rest” vs. “Ranch, 8H Rest”, and “Ranch, 8H Rest” vs. “Ranch, 0H Rest”.

Several β-binomial regression comparisons were made to evaluate the effects of cattle source and rest time (Figure 7). For all comparisons, each of the top 9 most abundant genera were part of the taxa that differed [*p* < 0.05, log2(FC) > 2 or log2(FC) < −2] between sampling times and treatment groups. When comparing AM-0H against Ranch-0H, 67 genera were identified in AM-0H calves whose change in relative abundance from the BL sampling time were significantly different from Ranch-0H calves (*p* < 0.05). In general, most of the taxa became less abundant in AM-0H calves. A notable exception was *Pasteurella*, which showed the most significant increase from the BL sampling time in AM-0H calves, and consistently remained elevated. Similarly, *Mannheimia* was consistently elevated in AM-0H calves at each time point. Other BRD-related genera, including *Histophilus, Moraxella*, and *Mycoplasma* had changes that were varied at time points, though *Moraxella* and *Mycoplasma* were reduced at most times for AM-0H calves compared to Ranch-0H calves. When AM-8H calves were compared to Ranch-8H, 77 genera were observed to change in relative abundance, with most changes showing reduced abundance. Most changes in taxa were not consistent across time, but several genera were shown to be continuously reduced across all time points, including *Acinetobacter*, *Alistipes*, *Atopostipes*, *Dietza*, *Jeotgalicococcus, Lachnospiraceae* NK3A20 and *Streptococcus*, while *Histophilus* and *Mannheimia* were increased at all time points. Similarly, *Mannheimia* was elevated at all time points in Ranch-8H, when compared to Ranch-0H, as was *Pasteurella*. There were 72 genera that differed in relative abundance in Ranch-8H calves compared to Ranch-0H calves, with most showing a trend of increased abundance. For AM-8H calves, 73 genera showed a change in relative abundance compared to AM-0H calves. *Aminobacter*, *Corynebacterium*, and *Lachnospiraceqae* UC6-006 were increased across time in AM-8H calves, while *Mannheimia* was reduced at all time points except for 1 day AU, and *Pasteurella* was reduced at all time points, compared the AM-0H group. In Table 1, BRD-associated genera that differed at each time point AU are listed.

**Table 1:** Number of time points (*N* = 5) when BRD-associated genera differed when comparing auction-market or ranch-sourced cattle, with (8H) and without (0H) rest-stop during transportation^a^.

Genera	AM-0H vs. Ranch-0H		AM-8H vs. AM-0H		AM-8H vs. Ranch-8H		Ranch-8H vs. Ranch-0H	
	Increase	Decrease	Increase	Decrease	Increase	Decrease	Increase	Decrease
*Histophilus*	2	2	3	1	5	0	1	2
*Mannheimia*	5	0	1	4	5	0	5	0
*Moraxella*	1	4	4	1	0	5	4	1
*Mycoplasma*	1	4	4	1	4	1	0	5
*Pasteurella*	5	0	0	5	2	3	5	0

a Data summarized from comparisons made in Figure 7. Cattle were transported 36 h, followed by assigned rest stop duration, and then an additional 4 h to a feedlot. AM, auction market-derived; Ranch, ranch-derived; 0H, 0 h of rest; 8H, 8 h of rest.

### Association of blood parameters to the top 9 genera

**Figure 8:**
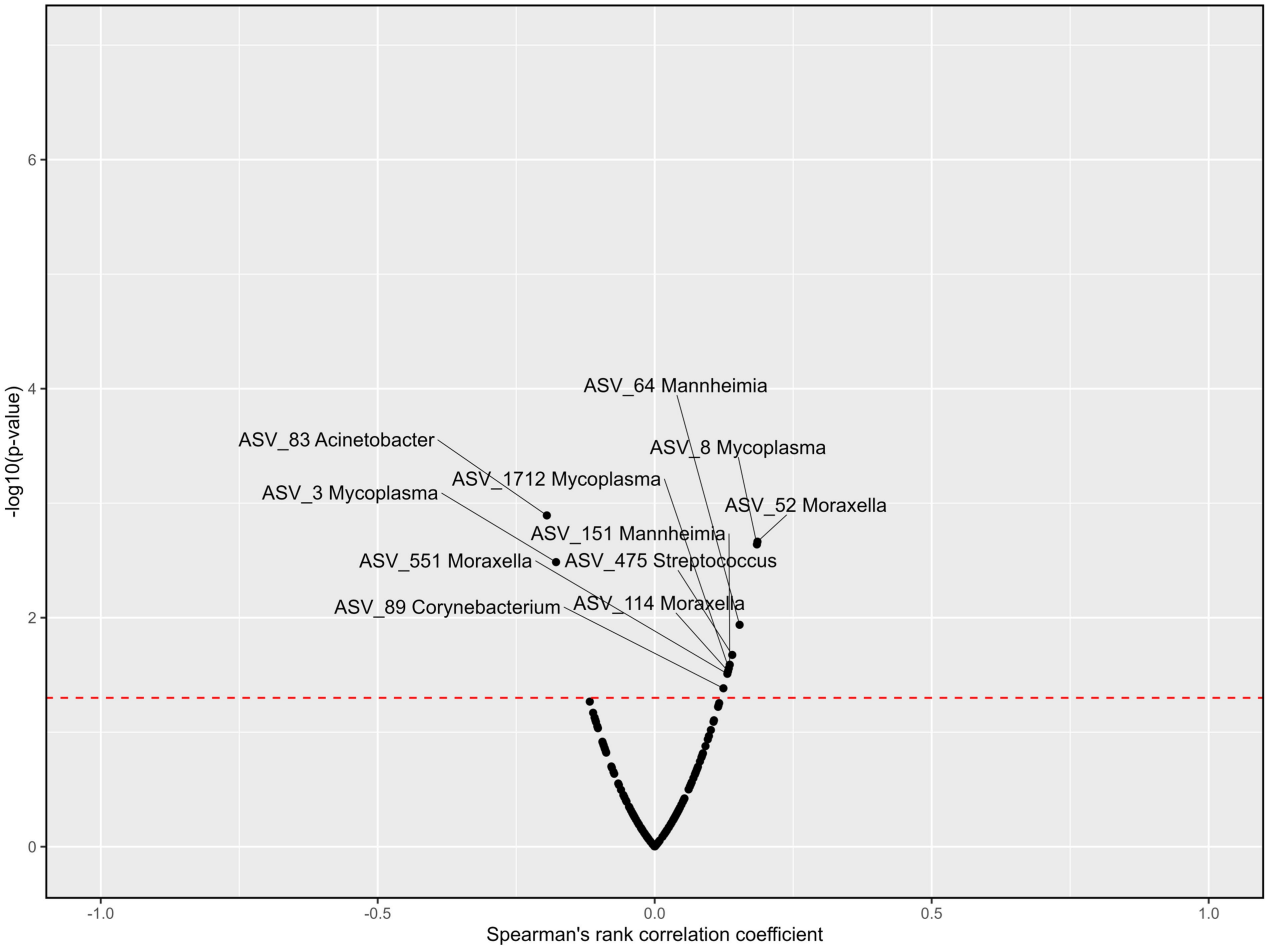
Volcano plots describing the correlations between ASVs within the nine most abundant genera and cortisol concentrations across all treatments and time points. Significance [−log10(value of *p*), y-axis] was calculated for the correlation of the counts for each ASV and the cortisol levels among the samples. Spearman’s rank correlation coefficient (r_s_, x-axis) assesses monotonic relationships of two variables, i.e., an r_s_ of 1 represents two variables with an identical trend of observations, and − 1 when the observations fully opposed. The red line represents the value of *p* threshold of 0.05. Genera with a correlation to cortisol levels that is *p* < 0.05 are labeled. Cortisol concentrations were previously reported by Meléndez et al. (2021).

**Figure 9:**
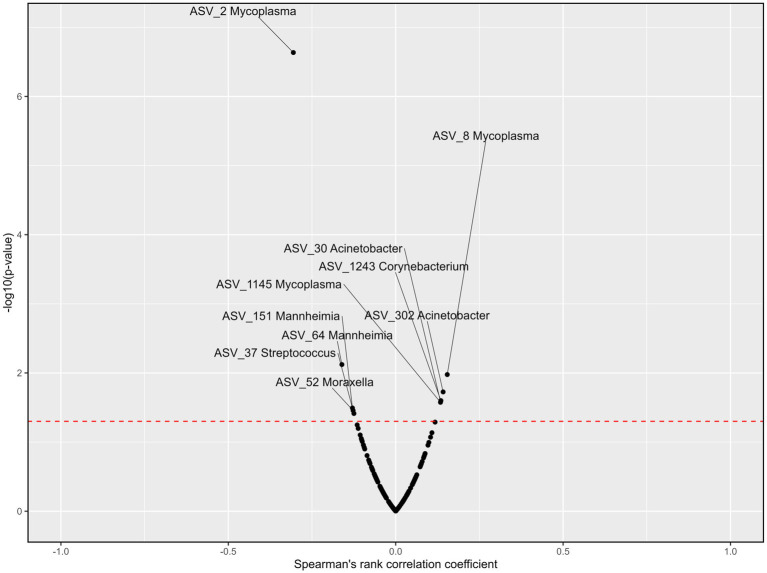
Volcano plots describing the correlations between ASVs within the nine most abundant genera and haptoglobin concentrations across all treatments and time points. Significance [−log10(value of *p*), y-axis] was calculated for of the correlation the counts for each ASV and the haptoglobin concentrations among the samples. Spearman’s rank correlation coefficient (r_s_, x-axis) assesses monotonic relationships of two variables, i.e., an r_s_ of 1 represents two variables with an identical trend of observations, and −1 the when observations fully opposed. The red line represents the value of *p* threshold of 0.05. Genera with a correlation to cortisol levels that is *p* < 0.05 are labeled. Haptoglobin concentrations were previously reported by Meléndez et al. (2021).

Six genera were correlated to blood cortisol concentrations across all treatments and time points (Figure 8). *Acinetobacter* was negatively associated with cortisol (r_s_ = −0.19, *p* < 0.01). Although *Mycoplasma* (ASV_3) was negatively associated with cortisol (r_s_ = −0.18, *p* < 0.01), *Mycoplasma* (ASV_8) was positively associated with cortisol (r_s_ = 0.13, *p* < 0.05). *Mannheimia, Moraxella, Streptococcus*, and *Corynebacterium* were also positively correlated with blood cortisol concentrations (r_s_ = 0.15, 0.19, 0.14, and 0.12 respectively; *p* < 0.05). Blood haptoglobin concentrations were correlated to six genera across treatments and time points (Figure 9). *Acinetobacter* and *Corynebacterium* were positively correlated with haptoglobin (r_s_ = 0.13 and 0.14, respectively; *p* < 0.05). Two ASVs of *Mycoplasma* were also positively correlated with haptoglobin (r_s_ = 0.15 and 0.13, respectively; *p* < 0.05), while one was negatively correlated with haptoglobin concentrations (r_s_ = −0.31, *p* < 0.001). *Mannheimia, Moraxella* and *Streptococcus* were also negatively correlated with blood haptoglobin concentrations (r_s_ = −0.16, −0.13, and −0.12 respectively; *p* < 0.05).

## Discussion

Understanding how transportation affects the bovine NP microbiota following introduction to a feedlot is important due to the fact that beef cattle are most susceptible to BRD during this period of time. The transportation of cattle to the feedlot has been associated with an increased risk of developing BRD (Taylor et al., 2010), and may be related to physiological changes resulting from stress, that allow for pathogen proliferation and infection (Storz et al., 2000). In addition to changes in pathogen prevalence, it is possible that alterations in other community bacteria affect BRD pathogen growth. Aerobic bacteria from the bovine respiratory tract have previously been shown to both enhance and inhibit the growth of *M. haemolytica* and *P. multocida in vitro* (Corbeil et al., 1985), thus the total NP microbiota may be critical to disease susceptibility. In the present study, it was determined that the NP microbiota of calves undergo numerous changes, both in community membership and structure, that were dependent on cattle source, rest stop duration, and transportation time.

### Microbial diversity of the nasopharynx

Alpha diversity metrics indicate the structure of an ecological community in terms of its richness (number of taxonomic groups present) and evenness (distribution of abundances; Callahan et al., 2017; Kers and Saccenti, 2022). Shannon’s index (α-diversity) estimates the diversity of taxa by taking into account both richness and evenness (Lemos et al., 2011), while the Bray–Curtis Metric (β-diversity) is used to evaluate dissimilarity in the microbial communities of samples (Kers and Saccenti, 2022). Diversity in the composition and structure of the NP microbial communities (β-diversity) was altered after feedlot placement and was mostly a result of less relatively abundant taxa dominating the population. This shift might have been due to several factors. It is likely that the feedlot was a source of bacteria that colonized the respiratory tract through aspiration, or airborne nutrients that may have promoted growth of certain bacterial species. In addition, the change in diet may have altered the gastrointestinal bacterial microbiota, which colonized the respiratory tract through oropharyngeal transfer (Hall et al., 2017). Indeed, several genera associated with the gastrointestinal tract did increase in relative abundance after unloading (e.g. *Bacteroides*, *Prevotella*).

All calves were administered tulathromycin after feedlot arrival, as part of a commonly employed management practice to mitigate BRD. While Holman et al. (2019) did not see an increase in diversity of the NP microbiota of calves administered tulathromycin, they did observe an increase in diversity in calves administered oxytetracycline. They attributed this to potential disruption of the NP microbiota caused by oxytetracyline inhibiting certain bacterial populations and allowing others to fill the niche and colonize. Interestingly, we have observed tulathromycin to reduce the total bacteria in the nasopharynx of cattle mainly through reduction of *Proteobacteria*, while still resulting in increased diversity (Amat et al., 2023). In the present study, *Proteobacteria* was also reduced after feedlot processing, indicating that this phylum may have increased susceptibility to tulathromycin.

### Microbial composition and structure of the nasopharynx over time

Overall, the most abundant phyla were *Proteobacteria*, *Mycoplasmatota* (formerly *Tenericutes*), and *Actinobacteria*. In agreement with our study, *Proteobacteria*, *Mycoplasmatota, Actinobacteria, Bacteroidetes*, and *Firmicutes* have been reported to be the most relatively abundant phyla in nasopharynx of early, or newly weaned, and feedlot placed cattle (Timsit et al., 2016b; Holman et al., 2017; Stroebel et al., 2018). *Mycoplasmatota* was the most relatively abundant phylum, and this phylum has been reported previously to be the most abundant phylum in the nasopharynx (Stroebel et al., 2018; Timsit et al., 2018) and trachea (Timsit et al., 2018) of feedlot cattle sometimes comprising more than 40% of the total microbial community. In contrast, *Firmicutes* accounted for 3.6% of the relative abundance. The relative abundance of *Firmicutes* in the nasopharynx of newly weaned and auction- or ranch-derived feedlot heifers was reported by Stroebel et al. (2018) to be only 3%, whereas Holman et al. (2017) reported it to be greater than 20%, showing that significant variation in the major phyla occur in cattle.

*Mycoplasma, Acinetobacter*, and *Corynebacterium* were the most relatively abundant genera and tended to increase after feedlot placement, which has been observed previously (Holman et al., 2017). These genera are often reported to be dominant in the nasopharynx of healthy feedlot cattle (Holman et al., 2017; Zeineldin et al., 2017; Holman et al., 2018; Stroebel et al., 2018) and once established may represent a state of a more mature and stable respiratory microbiota in feedlots. For all cattle groups, genera most often associated with BRD (*Mannheimia*, *Pasteurella*, *Histophilus*, and *Mycoplasma*; Booker et al., 2008; Confer, 2009) were part of the 9 most relatively abundant genera. While these bacteria are part of the normal NP microbiota and colonize shortly after birth (Lima et al., 2016), they are opportunistic pathogens and are increased in the NP and lungs of cattle with BRD (Timsit et al., 2018). In healthy cattle, it has been observed that *Mannheimia* and *Pasteurella* tend to decrease in abundance over time in a feedlot (Holman et al., 2017), particularly when calves are administered metaphylaxis antimicrobials at entry (Holman et al., 2019). Interestingly, *Pasteurella* generally increased and remained elevated throughout the study. Perhaps this was due to calves being colonized by tulathromycin-resistant *Pasteurella*, which were not inhibited by tulathromycin administration. In support of this, we have previously observed over 70% of *P. multocida* from feedlot calves to display resistance to tulathromycin (Timsit et al., 2017) and have shown that antimicrobial-resistant *P. multocida* clones can be selected by antimicrobial administration and colonize pen mates in feedlots (Guo et al., 2020).

*Bifidobacterium* tended to mostly be increased at time points subsequent to loading for transportation, suggesting that this genus may colonize the upper respiratory tract as a result of environmental exposure. While typically associated with the gastrointestinal tract of mammals, *Bifidobacterium* has repeatedly been identified in the respiratory tract of cattle (Holman et al., 2019). Thus, digesta of the gastrointestinal tract, or feces and manure, may be a source of this genus. *Bifidobacterium* spp. are used as probiotic agents and have been investigated in humans to prevent or treat upper respiratory tract infections (Popova et al., 2012). Whether *Bifidobacterium* spp. in the upper respiratory tract of cattle confers a similar beneficial effect is unknown. Other gastrointestinal bacteria have routinely been detected in the NP of cattle (Holman et al., 2017; Stroebel et al., 2018). Taxa within *Prevotellaceae* were also shown to mostly increase subsequent to feedlot unloading. *Prevotella* spp. are obligate anaerobes that have been identified from both nasopharynx and tracheal samples obtained from cattle (Lima et al., 2016; Timsit et al., 2018; Klima et al., 2019). The relative abundance of this genus in the nasopharynx has been reported to be similar among healthy or pneumonic dairy calves (Lima et al., 2016). Tracheal samples from healthy feedlot cattle have also been noted to be enriched with *Prevotella* compared to those with BRD (Timsit et al., 2017). *Prevotella* is typically the most abundant genus in the rumen (Henderson et al., 2015), and *Prevotella* spp. respond to the acidity of the rumen, becoming more enriched when a grain-based diet is fed to the cattle (Petri et al., 2013). It is therefore plausible that the increased abundance of *Prevotella* was associated with changes in the diet after feedlot placement, though whether colonization is transient requires further evaluation.

### The effect of rest stop duration and cattle source on bacteria of the nasopharynx

*Filobacterium* was part of the top 9 abundant genera. This genus has more recently been classified and has been linked to respiratory disease in rodents (Ike et al., 2016). It has also been detected in high abundance in the nasopharynx of healthy feedlot cattle (Holman et al., 2018), and so the biological significance of this genus in the bovine respiratory tract remains unclear. When ranch calves were rested for 8H during transportation, it resulted in an increase in relative abundance of *Mannheimia*, as well as *Pasteurella*, another genus associated with BRD. Interestingly, the same result was not observed when comparing calves processed through an auction market that were given 8H of rest compared to those that did not have rest. While difficult to explain, it could be that the impact of the auction market itself on the NP microbiota was greater than the rest period. For both sources of cattle, an 8H rest period resulted in a prolonged increase in *Streptococcus*. Members of this genus are known to encode sialidases which can degrade mucus glycans (Xu et al., 2008, 2011), impairing respiratory health (Parker et al., 2009; King, 2010; Lewis and Lewis, 2012). These glycans play important roles in host mucosa surface integrity and pathogen colonization resistance. Whether alterations in glycan-degrading bacteria leads to compromised mucosa would be an interesting concept to study further.

When comparing auction market calves to ranch calves with similar rest times, there were stable increases in *Mannheimia* at all time points, and a strong increase in *Pasteurella* for the AM-0H group relative to the Ranch-0H group. The structure of the microbiota also differed according to cattle source. This is in contrast to a previous study that did not detect differences in diversity or composition of NP and tracheal bacteria in cattle transported to an auction market, vs. those that were directly transported to the feedlot from the ranch (Stroebel et al., 2018). Several factors may have led to these differences, including the source of calves, duration of transportation which was less (approximately 2.5–3H from auction market to feedlot) than our study.

The genus *Lactobacillus* was reduced in NP samples of auction market calves compared to ranch calves, and 8H rested calves compared to no rest calves. Among commensal bacteria, *Lactobacillus* has been shown to have a potential role in providing colonization resistance against respiratory pathogens. Specifically, *Lactobacillus* species have been isolated from the respiratory tract of cattle that can inhibit *M. haemolytica* both *in vitro* (Amat et al., 2017, 2019) and *in vivo* (Amat et al., 2020). This genus has also been found to be increased in healthy cattle, compared to those diagnosed with bronchopneumonia (Timsit et al., 2018). Whether the reduction of important commensals increases the risk of BRD is still not well described, but it was apparent that the reduction of *Lactobacillus* coincided with increase in some BRD-associated genera.

### The effect of rest stop duration and cattle source on BRD-associated bacteria

Bacteria most often associated with BRD cases include *Pasteurella*, *Mannheimia*, *Histophilus*, and *Mycoplasma* (Booker et al., 2008; Confer, 2009; Timsit et al., 2017), with *Mannheimia* and *Pasteurella* being the primary cause of fatal secondary infections after feedlot arrival (Malmuthuge et al., 2021). In addition, *Moraxella* has recently been observed as a potential risk factor for BRD in received cattle (McMullen et al., 2020) thus these genera were used to summarize the impact of treatments on BRD-associated bacteria. Overall, there was a clear trend that placement in an auction market resulted in increased relative abundance of these genera at most time points after transportation. This was particularly the case for *Mannheimia* and *Pasteurella*. Rest during transportation trended toward an increase in relative abundances of BRD-associated bacteria, particularly for *Moraxella*, but the other bacteria varied more with cattle source. Ranch-sourced cattle with rest during transportation had increased relative abundances of *Mannheimia* and *Pastuerella*, with relatively fewer *Mycoplasma*. In contrast, auction-market sourced cattle with rest during transportation had higher *Mycoplasma* and *Histophilus* abundances relative to the un-rested group, but lower *Mannheimia* and *Pastuerella* abundances. The majority of *Mannheimia* and *Pasteurella* have previously been shown to belong to *M. haemolytica* and *P. multocida*, respectively (Malmuthuge et al., 2021), likely suggesting an increase in these species in our studies. However, *Mycoplasma* comprises multiple species that colonize the bovine respiratory tract, which may have resulted in the variation observed for its relative abundance. For example, *M. bovis* has been reported to increase in the upper and lower respiratory tracts of calves diagnosed with BRD, compared to their healthy counterparts, while *Mycoplasma dispar* was more abundant in healthy cattle (Timsit et al., 2018). Others have reported enrichment of *Mannheimia* and *Pasteurella* in BRD morbidities (Timsit et al., 2018; Centeno-Martinez et al., 2022), and *M. haemolytica* colonization at feedlot entry has been associated with increased risk of BRD (Noyes et al., 2015). This suggests that auction market exposure and rest stop for ranch-derived cattle may be important risk factors for BRD, based solely on increased abundance of BRD-associated genera in the upper respiratory tract. There were too few animals in our studies to support this however, as BRD morbidity was low. Larger scale studies are therefore needed to further evaluate how these transportation and intermediary site (auction market and rest stop facilities) variables impact cattle health and welfare.

### The correlation between physiological stress parameters and bacteria of the nasopharynx

Serum cortisol concentrations were collected as an indicator of acute stress, to see whether the top nine most abundant genera responded to physiological changes (increased blood cortisol concentrations) in the host related to stress (transport, handling, novel environment). While variation was observed in ASVs for *Mycoplasma*, all *Mannheimia, Moraxella* and *Streptococcus* ASVs that had significant correlations with cortisol, were positive. Recently, multiple studies have reported that increased cortisol concentrations were associated with oral (Duran-Pinedo et al., 2018), nasal (Zhao et al., 2020) and gut (Mudd et al., 2017) microbiota perturbations. Zhao et al. (2020) also showed that long-distance transportation of donkeys increased cortisol concentrations and altered the nasal microbial population, though correlations were not reported. Infection from members of the Streptococcus genera have also been reported with elevated host cortisol concentrations in swine (Neila-Ibáñez et al., 2023) and humans (Beran et al., 2011). The stress hormone norepinephrine has also been shown to augment virulence in *Streptococcus pneumonia* (Sandrini et al., 2014), potentially inducing translocation from the upper respiratory tract to the lung, and potentiating infection (Alghofaili et al., 2021). We hypothesize that it is possible that similar host stress factors affect the virulence of BRD pathogens, or in the case of *Streptococcus* in the bovine respiratory tract, potentially modulate bacteria that can impact host defenses. The mechanisms by which these occur is worthy of further study to elucidate BRD pathogeneses.

Haptoglobin was measured as an indicator of infection and inflammation (Moisá et al., 2019). While haptoglobin concentrations were correlated to several taxa, it was difficult to see any clear trends as bacteria were both positively or negatively correlated with haptoglobin across treatments and time points. It is possible that the sampling time points were too infrequent to identify meaningful associations between haptoglobin and the microbiota. In a recent study, it was also shown that transportation affected the expression of adrenergic receptor genes in blood leukocytes of calves (Malmuthuge et al., 2021), perhaps indicating that catecholamines may be more likely to reflect changes in the respiratory microbiota, as a result of stress. It is also possible that evaluating local host stress responses directly in the respiratory tract may be more accurate in detailing how stress impacts the microbiota and potential disturbances to the mucosa.

## Conclusion

This is the first study to evaluate the effect of rest and cattle source on the respiratory microbiota of cattle, using appropriate control groups for comparison. It was evident that the bovine respiratory microbiota underwent a number of significant and relatively rapid changes in structure and composition when cattle were transported and placed in a feedlot. Microbial diversity was altered after feedlot arrival, likely due to introduction of novel bacteria from the feedlot environment and a change in diet causing shifts in rumen bacteria that reached the upper respiratory tract through regurgitation. Throughout feedlot placement, the respiratory microbiota was dynamic, with both commensal and pathogenic genera fluctuating. However, several key genera became dominant and established a more stable population over time. Although, there were no clear trends showing a relationship between haptoglobin and bacteria, cortisol was positively associated with *Corynebacterium*, *Moraxella*, *Streptococcus* and the BRD-associated genus *Mannheimia*. It was notable however, that when cattle were off-loaded for a rest during transportation, several key bacterial genera associated with BRD experienced variation compared to animals given no rest stop. This variation trended toward increased abundances for most sampling times after feedlot placement. Similarly, more sampling time points had elevated levels of BRD-associated genera when auction market were compared with ranch-direct calves. This suggests that off-loading cattle for a rest, and putting calves through an auction market, may be risk factors for BRD, based solely on increased abundance of BRD-associated genera in the upper respiratory tract. However, additional studies are needed to further define how these transportation variables impact cattle health including the combination of varying transportation and rest stop durations.

## Data availability statement

The datasets presented in this study can be found in online repositories. The names of the repository/repositories and accession number(s) can be found at: https://www.ncbi.nlm.nih.gov/, BioProject accession PRJNA916751.

## Ethics statement

The animal studies were approved by Animal Care Committee, Lethbridge Research and Development Centre. The studies were conducted in accordance with the local legislation and institutional requirements. Written informed consent was obtained from the owners for the participation of their animals in this study.

## Author contributions

TA, KS-G, and YN conceived and designed the experiment. DM and MU collected samples and performed lab work. MW, MU, and TA performed 16S rRNA gene sequencing analysis. MU and TA wrote the manuscript. All authors contributed to the article and approved the submitted version.

## Funding

This work was supported by the Beef Cattle Research Council (Project #ANH.22.18) and Results Driven Agriculture Research (RDAR, Project #2020F046R).

## Conflict of interest

The authors declare that the research was conducted in the absence of any commercial or financial relationships that could be construed as a potential conflict of interest.

## Publisher’s note

All claims expressed in this article are solely those of the authors and do not necessarily represent those of their affiliated organizations, or those of the publisher, the editors and the reviewers. Any product that may be evaluated in this article, or claim that may be made by its manufacturer, is not guaranteed or endorsed by the publisher.
