# Supplementary material for: Auction market placement and a rest stop during transportation affect the respiratory bacterial microbiota of beef cattle
Source: Front Microbiol. 2023 Sep 22;14:1192763. doi: 10.3389/fmicb.2023.1192763 (PMC10556482; doi:10.3389/fmicb.2023.1192763)
Supplement: Supplementary file 1 [file Image_1.pdf]

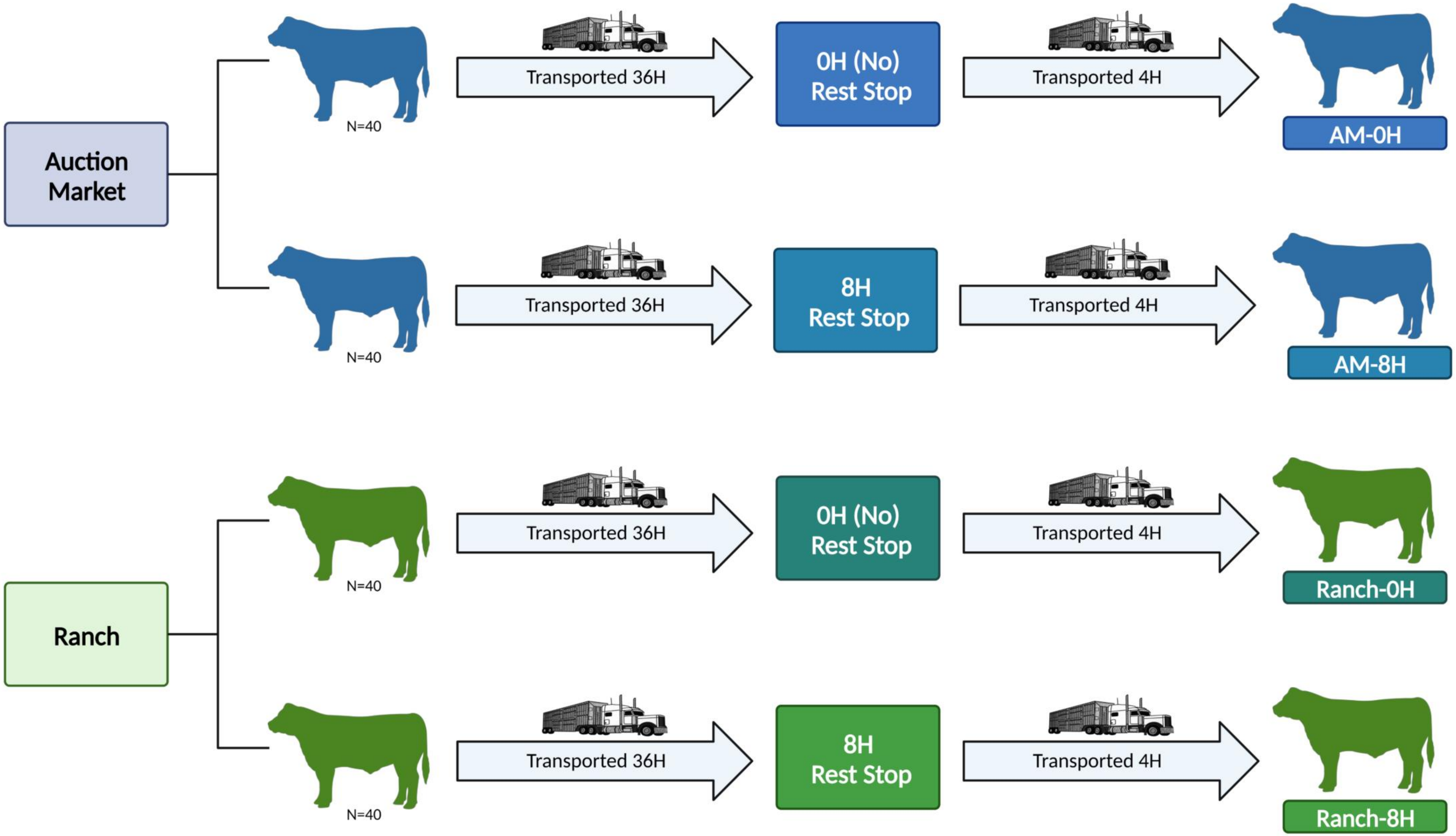

Supplementary Figure 1: Experimental design. One hundred sixty crossbred steer calves were sourced from a single farm in southern Alberta and transported to the Lethbridge Research and Development Centre (LeRDC). Calves were randomly divided into two groups: those shipped to an auction market prior to transportation (Auction Market, AM), or those directly transported without auction market placement (Ranch). Within each group, calves were randomly assigned to two rest stop times, which included 0H or 8H of rest, following 36 H of transportation. These four treatment groups are labelled by source and rest time: AM-0H, AM-8H, Ranch-0H, and Ranch-8H. Calves were transported for 36 H, followed by the assigned rest stop time (0 or 8 H), and transported another 4 H for delivery to the LeRDC feedlot. There were 40 calves in each treatment group, housed in 4 pens (10 animals per pen).
